# An industry-level analysis of the post-Brexit and post-Covid 19 Ro-Ro ferry market and critical maritime freight transport links between the UK and the EU

**DOI:** 10.1186/s41072-022-00127-4

**Published:** 2022-12-09

**Authors:** Dimitrios Paraskevadakis, Adeyeri Ifeoluwa

**Affiliations:** Liverpool Logistics Offshore and Marine Research Institute (LOOM), School of Engineering, Liverpool John Moores University, Merseyside, UK

**Keywords:** Short sea shipping, Covid-19, Brexit, Freight ferries, Ferry routes, Global trade, Intermodal transport, Logistics

## Abstract

Evaluating supply chain system improvements and international economic integration patterns to changes in expenditure requires urgent attention from short to long-term supply chain disruption review. Following the United Kingdom's exit from the European Union, the UK and the global economies have been affected. Brexit has affected sectors that rely on cross-border commerce with EU countries, while services that require face-to-face interaction have been negatively impacted by Covid 19. For example, the Roll on Roll off (RoRo) ferry sector has suffered tremendously from the Covid 19 pandemic, which restricts people's travel, and Brexit, which results in the UK having a geographical barrier with the rest of the EU continent. The study examines the ferry RoRo market, maritime links, and its viability in the face of Brexit and the Covid issue between the United Kingdom and short-sea shipping connections with Ireland and continental Europe. Understanding the market is critical since roll-on roll-off (Ro-Ro) traffic plays a significant part in the movement of products between the UK and the EU. Adopting a method mapping provided a defined research paradigm for this study. Mixed-method design technique, a branch of multiple methods research, was employed, resulting in a better understanding of the research topic. The findings demonstrate that nations and the RoRo freight supply chain network have been significantly impacted. However, continuous measures are implemented to ensure continuous freight movement. The study obtained data from 14 respondents and was statistically analysed. The results demonstrate that an organisation's capacity to manage these disturbances significantly impacts its survivability. It also indicates that the government's awareness of the implications of Brexit and Covid 19 significantly determines the impact on organisations. This research concludes that the impact on organisations is minimal, notwithstanding the effects of Brexit and Covid. However, owing to uncertainties, continuous methods for continuous freight transit should be implemented, together with an adequate flow of information.

## Introduction

Maritime transport and logistics are critical when evaluating countries' economic performance. The expansion of the economies of the United Kingdom (UK) and the Republic of Ireland (ROI) is heavily driven by trade, with logistics being the most crucial factor (Tetlow and Pope 2020). Compared to other countries, these countries import and export of products and services to and from the EU are high. In a national referendum on June 23, 2016, the British opted to leave the EU, commonly termed Brexit. The referendum's outcomes have produced political, social, and economic uncertainty with far-reaching consequences. It has ushered in a new Europe in which old commercial ties, methods, and protocols, which have evolved since 1973, are antiquated and redundant (Brady, 2021). As a result, businesses in all of these nations suffer negative consequences. While Brexit remained a source of uncertainty, the global pandemic known as Coronavirus (Covid 19) caused increased alarm among businesses and has remained a big concern.

According to UNCTAD (2020), immediate attention is required from short to long-term evaluation of these changes, evaluating an improvement in the supply chain system and international economic integration patterns to changes in expenditure. Following the UK's exit from the EU, the UK economy has been affected and the world economy at large; Brexit has affected sectors that rely on cross-border commerce with EU countries, while Covid 19 affects services that require face-to-face interaction (Tetlow and Pope 2020). The Covid 19 epidemic restricts people's migration, and Brexit, which results in the UK having a geographical barrier with other EU countries, have had a significant impact on the RoRo ferry sector (Vega et al. 2018). The UK, to statistics, relies significantly on ferry traffic for a large chunk of its trade. The ferry trade includes food supplies. While social distancing may be achievable in short-sea routes, it is almost impossible when these ferries have to ply longer routes that might require overnight accommodation (Vickerman 2021).

Additionally, in the past, ROI relied on the UK landbridge to export and import commodities into and out of the European continent. Compared to the more cost-effective and less frequent direct continental route, this provides a rapid, dependable, and secure service along the UK landbridge route. Brexit has substantially impacted the relative competitiveness of marine services along these maritime routes (Vega et al. 2018).

However, Brady (2021) pointed out that while Brexit and Covid 19 pandemic presents an array of challenges for traders, it is yet another example of a disruption many must manage in supply chain management (SCM). Modern supply chains are subjected to many risks and impediments that jeopardise their ability to consistently meet or exceed performance expectations. As a result, management teams must be aware of potential disruptions that could affect the planned performance levels of their supply chains and build an organisation with the appropriate culture and practices to deal with disruptions proactively.

Roll-on roll-off (Ro-Ro) traffic plays a significant role in transporting goods between the UK and the EU. As a result of Brexit and Covid, more robust strategies are required to help coordinate national policies in the transport and logistics sector by removing barriers to growing trade, enabling and ensuring the continued movement of freight across countries via designated vital routes for supplies. Trade tariffs, border controls, customs clearance, and freight and passenger traffic checks have harmed logistical efficiency and the present supply chain. There is a need to monitor the industry due to the uncertainty and a sense of urgency to minimise by facilitating freight transportation services, logistics, and commerce through existing regional logistics gateways (Paraskevadakis 2020).

Various literature has given several presumptions of the impact of Brexit on the RoRo industry. Nevertheless, the sudden addition of the Covid-19 pandemic has added to the effects. In addition, there have been changes in RoRo traffic due to the strategies implemented by RoRo firms to manage disruptions.

## Background to RoRo ferry shipping

In the global shipping industry, ferries are remarkably exceptional because they operate only in a closed market with little or no competition, and their earnings are much more stable than in the volatile global shipping sectors. In addition, ferries tend to be more regional because of the unlimited demand for their services in inland waterways for the movement of passengers, cars, lorries, and trailers (Poulsen 2019). Talley (2021) describes ferries as passenger vessels that operate on a fixed time and fixed water route to transport passengers and their luggage, which may occasionally include automobiles via roll on—roll off ferries, or cargo transported in packages or containers. Poulsen (2019) argues that maritime investors have shown little interest in it despite the ferry industry's importance and adaptability, preferring the cargo and heavy shipping industries. Though ferries were initially thought to be designed for a single route, the 1980s demonstrated that ferries could provide additional services by diversifying their offerings to include bars, casinos, show lounges, swimming pools and restaurants.

Several types of ferries, including hovercrafts, catamarans, hydrofoils, and monohull ferries, have an increased cargo capacity than catamaran ferries. Additionally, the double-ended ferry can switch bows and sterns without turning while operating in a marine terminal (Talley 2021). This research is specifically focused on the Ro-Ro ferry. According to Bureau Veritas (2021), Ro-Ro ships, also known as rolled-on and rolled-off ships, have a loading ramp to facilitate the loading and unloading of wheeled vehicles.

The purchase of RoRo shipping services is significantly different from other marine sectors due to its requirements and much higher demand elasticity since it faces stiff competition from land-based modes of transport. RoRo shipping suppliers always seek to meet the needs of shippers, and their provision of RoRo services is heavily impacted by the shippers' requirements that 'define' their operations. Furthermore, RoRo services are comparable to liner services in that they provide frequent, scheduled, and conventional seaborne transit between predetermined ports of call (Christodoulou and Kappelin 2020). According to Brambilla and Martino (2016), ferry firms generate more revenue transporting cargo than transporting passengers, increasing demand for roll-on–roll-off ferries. The RoRo firms compete by increasing service flexibility, adding leisure facilities on board to attract more customers, and catering to the client's preferences. This competes heavily with cruise lines, as RoRo ferry companies place a high premium on passenger expectations (Kizielewicz et al. 2017).

## Impact of Brexit and Covid on the RoRo freight transportation between the UK and the EU

International maritime trade is critical for all nations because it enables economies of scale to create worldwide revenues, accelerate investment recovery, learn from exporting, and enhance efficiency and effectiveness. Amongst nations, each one's growth has the potential to have a good or bad effect on the other. Numerous disruptive factors such as economic (population, (de-) globalisation, re-shoring), political (e.g., tariffs and trade tensions), climate, advanced technologies, infrastructure development, new regulations, digitalisation, and the growth of e-commerce all have the potential to affect the outlook for any segment of a nation's maritime sector (Sys and Vanelslander 2021). According to Román (2017), freight transport is critical to a country's productive structure in terms of logistics and economic development in general; the deepening of the economy's internationalisation increases its relevance even more.

Since commodities are seldom consumed in the exact location as they are manufactured, a process of many interconnected decisions must be taken to guarantee that these goods reach their destination. Perhaps the most critical aspect of these selections is the method of transport chosen. Thus, thoroughly understanding the factors influencing this choice is critical for evaluating freight transportation strategies. RoRo freight transport is widely utilised in the United Kingdom, Republic of Ireland, and continental Europe. While the UK is a significant trading partner for ROI, as a peripheral island nation, it also acts as a critical transit network for access to European markets. Likewise, UK's import and export with the EU as a trade partner is high. Historically, Irish traders have used the UK's road network to carry products between Ireland and mainland Europe, thus utilising the UK as a bridge (Brady 2021).

With the UK's recent exit from the European Union, there have been significant worries about the future relationship between the UK and the EU as a trade partner. These nations have experienced substantial uncertainty over the possible impact of the UK-EU trade relationship's future development within their connection, with further consequences for the supply and demand for marine freight services from and to Ireland. Vega et al. (2018) mentioned there would be crucial difficulties in this group of states after Brexit which subsequently necessitated new plans for resolving uncertainties. Additionally, freight movements have benefited from the UK landbridge network's fast and economical service, backed up by a high degree of dependability and security. Although, marking the end of the transition period on December 31, 2020, Brexit resulted in customs and other inspections on goods entering, leaving, or transiting the UK. However, delays and congestion on the UK landbridge for commerce with continental Europe were anticipated prior to Brexit, prompting the UK, EU, and Ireland to propose early measures to ensure uninterrupted freight flow. While the UK and EU attempt to reach an agreement on a trade deal, Ireland attempts to find alternatives to the UK landbridge. The UK landbridge provides Irish exporters with very competitive and efficient service in terms of frequency and transit times, as well as a high level of dependability and security, at a little higher cost than alternative direct routes to continental Europe (Vega and Evers 2016). Nonetheless, Ireland is forced to function in the alternative by using direct routes to Europe to circumvent the customs rules imposed on the UK landbridge.

Consequently, the Covid 19 pandemic affected worldwide consumer behaviour by altering purchasing timing, breadth, and volume. Due to border controls arising from various sanitary measures, lengthy lines of trucks formed, causing delays in freight transit and eventually leading to driver unavailability (Tardivo et al. 2021). Brexit and Covid 19 are expected to result in modal and route adjustments, such as RoRo traffic on the UK landbridge moving to other modes of transportation and a move away from road-based freight transit toward more cost-effective modes such as Short Sea Shipping (SSS), and likewise increase in capacity (Vega et al. 2018).

The maximum available lane metres method is used to assess ferry and RoRo vessel capacity. A lane metre is a segment of a deck that is one lane wide and one metre long. As liner ships, Ro-Ros rarely have a fixed and precise maximum capacity. Instead, the capacity of RoRos and ferries is controlled by the cargo mix concerning moveable decks, internal ramps, lane heights, and other factors that may restrict the quantity of cargo that a vessel can carry (Styhre 2010). As a result of these anticipated severe changes, mathematical models must be employed to analyse the changing logistics capacity and increased traffic congestion that the maritime transport and logistics network may suffer in the future due to continuous uncertainty.

## Political and geographical economy of Ro-Ro freight transport following the impact of Brexit and Covid 19 pandemic

Collaboration and coordination across countries and channel partners (suppliers, intermediaries, third-party service providers, and customers) are critical components of supply chain management. Planning and management of all activities related to sourcing and procurement, conversion, and all logistics management operations are required for successful supply chain management (Anca 2019). Due to the rapid cargo handling and high frequency of departures, roll-on-roll-off (RoRo) shipping provides a maritime segment that is integrated into an intermodal transport system without difficulty. It contributes to sustainable transportation networks by eliminating the need for cargo to be transhipped in ports; instead, freight is 'rolled' to and from the sea on its wheels. The purchase of RoRo shipping services is significantly different from other marine segments due to its much higher demand elasticity since it faces stiff competition from land-based forms of transport (Christodoulou and Kappelin 2020).

According to Roscoe et al. (2020), geopolitical crises can dramatically disrupt supply networks. Supply chains frequently cross political borders, putting buyers and suppliers at risk of geopolitical disturbances. Supply chain uncertainty is characterised as risks that can arise in a global supply chain network, resulting in either positive or negative effects. The Brexit, or the United Kingdom's exit from the European Union, and the Coronavirus (Covid 19) have been two major disruptions to the UK economy and the rest of the world. Brexit has had a detrimental effect on sectors that rely on cross-border commerce with the EU, most notably Ireland, which is entirely reliant on the UK network, while coronavirus damages services that require face-to-face contact, with 69 per cent of the country expected to be affected (Tetlow and Pope 2020). Both significantly impact the Ro-Ro ferry industry, which provides both tradeable and non-tradeable services. In recent times, there have been concerns over these changes, given that UK Ferry services link to the European Continent and Ireland. The following section analyses the political, geographical, and economic aspects of RoRo freight flows in the United Kingdom, Europe, and the Republic of Ireland, particularly emphasising the impact of Brexit and the Covid 19 pandemic.

Commodities in one region must be processed, sorted, or consumed in another region, which necessitates freight transit. Freight transportation is, therefore, an example of what economists call a derived demand, as it is not necessary but is utilised to fulfil another demand. Private sector firms such as merchants, manufacturers, and processors supply freight transportation demand. However, such businesses are ultimately responding to customer demand for items and managing returns of undesired or defective goods and waste materials such as packaging for recycling or disposal (GO-Science 2019).

Reis (2019) has developed various integrated transport chain concepts, including intermodal, combined, co-modal, and, more recently, synchromodal transport. Integration includes the deliberate coordination and alignment of transport agents to optimise the transport service's performance. Integration has a significant role in the operation of freight transport systems. Private sector companies almost exclusively provide freight transportation and logistics services, investing heavily in fixed infrastructure such as ports, rail terminals, and distribution centres, as well as mobile equipment such as trucks, vans, forklift trucks, ships, and railway locomotives and waggons (Román 2017).

Intermodal or multimodal freight is an alternative to consigning goods for the entirety of their voyage via a single mode, of which RoRo services are a crucial component (Lowe 2005). RoRo services, which move cargo on trailers rolled on and off a ship without a crane, provide frequent marine transport linkages between the United Kingdom, the continental European mainland, and Ireland (GO-Science 2019).

## UK RoRo freight transport

Freight transport services help the overall running of the UK economy, especially the supply of products, which is crucial for the industry, and generating jobs. Freight transport can be broadly classified according to its origin or destination within the UK (domestic freight) or other countries (international freight). International freight passes through a port, airport, or the Channel Tunnel, while international freight transit in Northern Ireland may also include movements across the land border with the Republic of Ireland.

Frequent maritime linkages between Great Britain and the European mainland and Ireland are provided by RoRo services, which are unitised goods in trailers rolled on and off a ship without using a crane. The UK RoRo market is often divided into the GB–Continent market and the United Kingdom- Ireland market. The latter is divided into the Northern Corridor (UK ports to Northern Ireland), the Central Corridor (Lancashire and North Wales ports to Dublin), and the Southern Corridor (South West Wales ports to southern Ireland, while the former is divided into the Dover Straits (between Dover and the Hauts-de-France Region4), the North Sea corridor (connecting GB ports within Thames to Forth range to the Near Continent, Scandinavia, and the Baltic), and the Western Channel (between GB ports in the Newhaven to Plymouth range to France and Spain) (GO-Science 2019).

According to De Lyon and Dhingra (2021), Covid-19 has wreaked havoc on the UK RoRo freight economy on a scale never seen before, resulting in an almost 20% reduction in business volume. Additionally, with the UK exit from the EU on January 1 2021. This has led to trade, investment, and people movement obstacles between the UK and the EU. As a result, the volume of products traded between the UK and the EU decreased drastically in January 2021, with only a minor recovery in February. Exports to the EU were 38% lower in January 2021, while imports from the EU were 16% lower (Sampson 2021). Trade with non-EU nations declined significantly, with exports falling by 8% and imports decreasing by 9%. Trade flows have slowed due to Covid-19 limitations, and some of the declines may be attributable to stockpiling in anticipation of Brexit (De Lyon and Dhingra 2021).

All major UK ports dropped unitised traffic, including roll-on/roll-off (Ro-Ro). This drop was most evident in Ro-Ro traffic, propelled by a 4.2 million-unit decline in passenger vehicle units, down 67% yearly. Ro-Ro traffic dropped by varying magnitudes, depending on the measure employed. In tonnage terms, excluding passenger vehicle numbers, Ro-Ro declined by 5% in 2020 compared to 2019. However, the drop was considerably more pronounced when measured in unit terms, i.e. the number of cars handled at UK ports. In 2020, unitised Ro-Ro traffic decreased by 31% to 12.3 million units. The primary reason for this steep reduction was the 4.2 million decrease in passenger cars, which is primarily linked to the foreign travel restrictions implemented in 2020 to contain the spread of Covid-19 (DfT 2021a).

Based on the volume of goods handled, the most prominent ports are operated mainly by British Port Association, Forth Ports, Peel Ports, PD Ports, Hutchison, and DP World. Smaller ports are usually under the control of trust or municipal entities. The major ports of Dover, the Port of Tyne, London, Milford Haven (all trust ports), and Portsmouth (a municipally controlled port) are notable exceptions (GO-Science 2019). Dover is the largest ro-ro port in the United Kingdom, with overall ro-ro trade tonnage growing significantly since 2012. Dover, the United Kingdom's port, carries 22% of ro-ro tonnage and 26% of unitised traffic (DfT 2021a).

Dover is a vital link in the UK's trade with continental Europe. Since the Single Market was established in 1993, the number of lorries utilising its routes has increased by 150%, with over 2.5 million travelling through each year. Ireland is also a significant trade route between the UK and the EU. There is a regular movement of vehicles across the Irish land border, which includes over 200 crossing sites without customs formalities and has changed substantially since the UK exited the EU. Similarly, RoRo travel over the Irish Sea between Dublin and Holyhead transports a sizable part of the commodities moving between the United Kingdom and Ireland (Owen et al. 2017). As a result, large amounts of UK Ro-Ro traffic (which amounted to 73% of total Ro-Ro tonnage and unitised traffic) are accounted for by EU traffic; because of this, EU transport trends influence the Ro-Ro cargo category (DfT 2021a).

Owen et al. (2017) demonstrated that the UK's commerce with the EU relies heavily on the continual flow of lorries over the English Channel or the Irish Sea through ferries or railways. Tables 1 and 2 below summarise active ro-ro freight transportation by the route.

**Table 1:** Route 2 by Port Group—Dover Strait and the English Channel

Dover Strait	English Channel
Channel Tunnel Dover Boulogne Dover Calais Dover Dieppe Dover Dunkirk Ramsgate Ostend	Newhaven Dieppe Newhaven Le Havre Plymouth Cherbourg Plymouth Roscoff Plymouth Santander Plymouth St Malo Poole Bilbao Poole Caen Poole Cherbourg Poole Santander Portsmouth Bilbao Portsmouth Caen Portsmouth Cherbourg Portsmouth Le Havre Portsmouth Santander Portsmouth St Malo Portsmouth Zeebrugge Southampton Rouen

*Source* Own

**Table 2:** Route 3 by Port Group—Irish Sea (including domestic routes to Northern Ireland)

Irish Sea	Irish Sea (domestic routes to Northern Ireland)
Bristol Dublin Fishguard Rosslare Heysham Dublin Holyhead Dublin Holyhead Dun Laoghaire Liverpool Dublin Liverpool Porto Mostyn Dublin Pembroke Rosslare Swansea Cork	Cairnryan Belfast Cairnryan Larne Fleetwood Larne Heysham Belfast Heysham Larne Heysham Warrenpoint Liverpool Belfast Stranraer Belfast Troon Larne

*Source* Own

De et al. (2021) estimate that 61% of companies, including those in the services sector, have encountered at least one Brexit-related difficulty. The most common concerns involve the border, with 37% of businesses reporting delays, 36% citing increased customs and administrative expenses, and 22% reporting regulatory checks. DfT (2021b) reported that between June 2020 and June 2021, overall tonnage dropped by 3% to 432.1 million tonnes, and total unitised traffic decreased by 9% to 18.5 million units.

Road transport accounts for the vast bulk of RoRo freight transport with little percentage transported by rail. Coastal RoRo commerce accounts for a sizable portion. Commerce flows between the United Kingdom and the Republic of Ireland. GB links to the island of Ireland from the west coast Scottish, English, and Welsh ports are significant producers of road traffic on both sides of the Irish Sea. These volumes are quantified in the following analysis. The units handled in the accompanying table are in thousands and comprise all flows between British ports and the island of Ireland. Total RoRo freight volume on these routes is 1.93 million units, accounting for 32% of all RoRo freight traffic at UK ports.

These data indicate that over 3 million trucks and automobiles utilised the Irish Sea RoRo services in 2019, together with over 5 million passengers. However, as a result of Brexit's influence. In 2020/2021, specific Irish Sea RoRo volumes are diverted due to new direct shipping routes that bypass GB ports and provide a direct connection for EU commerce between Dublin, Rosslare, and Cork.

Additionally, Logistic UK (2021) suggests that the UK is facing a shortage of about 90,000 HGV drivers, putting an unsustainable strain on merchants and their supply chains due to the Covid-19 epidemic and Brexit. Driver training and testing were suspended for more than a year due to the pandemic, and only 600 EU drivers returned home throughout the pandemic following the transition period's conclusion, resulting in a scarcity of HGV drivers. Meanwhile, there have been modifications to the flow of products from and to the UK via roll-on roll-off. Before the items reach the departure port, a combined safety, security, and customs declaration must be performed. Similarly, customs declarations are made, and any applicable customs duty, excise duty, or VAT is paid (HMRC 2019).

## Continental EU RoRo freight transport

The European continent heavily relies on seaports for commerce with the rest of the world and inside its Internal Market. Ports ensure the EU's territorial continuity by connecting periphery and island territories via regional and local marine trade. In addition, they serve as hubs from which the trans-European network's multimodal logistic flows may be organised, utilising SSS, rail, and inland waterways connections to minimise road congestion and energy consumption (Pastori 2015). Although the European ferry industry is rigidly segmented into North and South networks, each has its unique market advantages and disadvantages. In specific locations, such as the Baltic Sea, the ferry and ro-ro markets have gained an advantage over their box-shaped competitors in recent years (Harbour Reviews 2017).

**Fig. 1:**
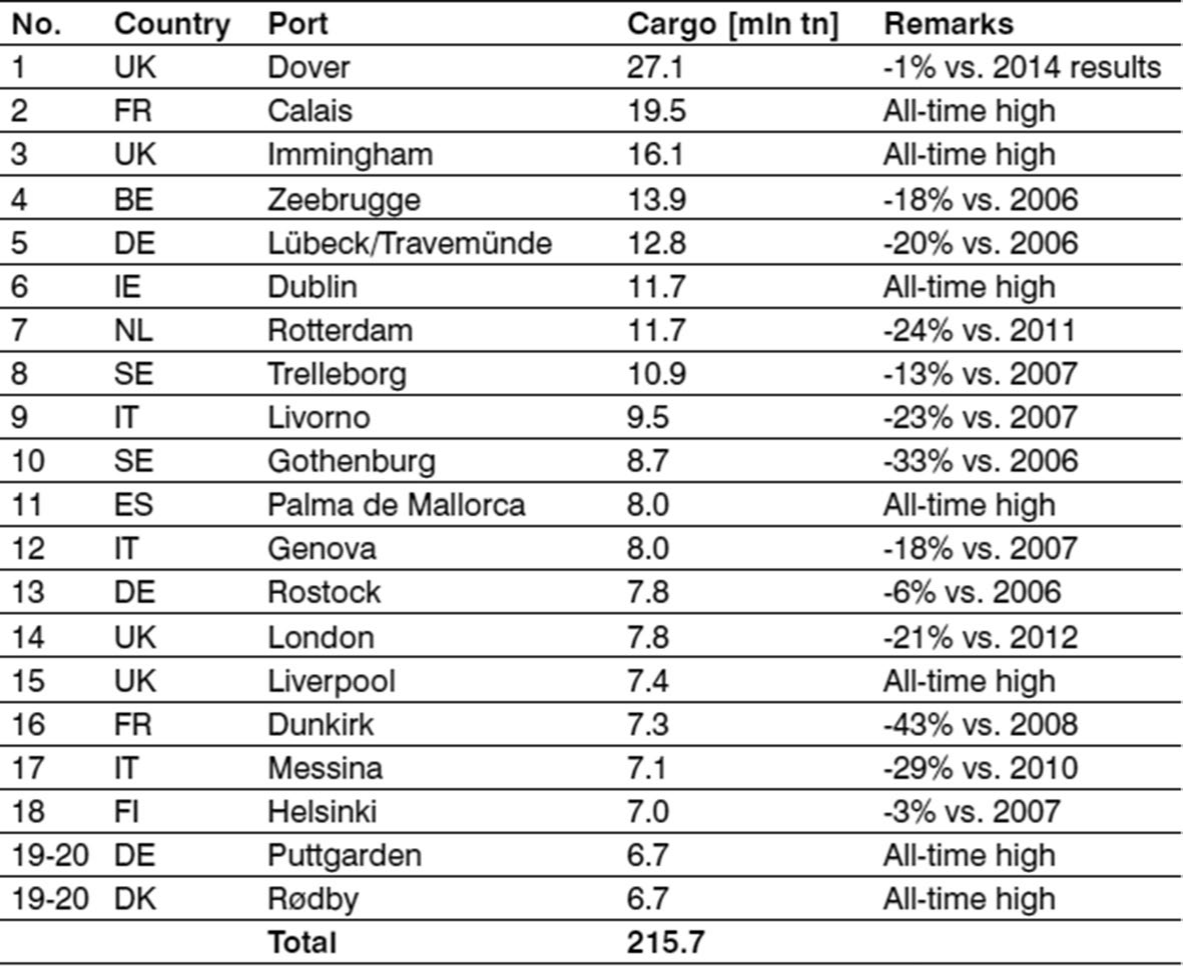
Europe’s Top 20 ferry/ro-ro freight ports in 2015. *Source* (Habour Reviews 2017)

Dover in the United Kingdom and Calais in France were the top two short seaports for roll-on/roll-off transport (RORO), with 27.1 and 19.5 million tonnes of RORO short sea traffic, respectively. These are significant ferry services operating across the English Channel. Figure 1 depicts Europe's Top 20 ferry/ro-ro freight ports in 2015, prior to the UK exit vote in 2016.

**Fig. 2:**
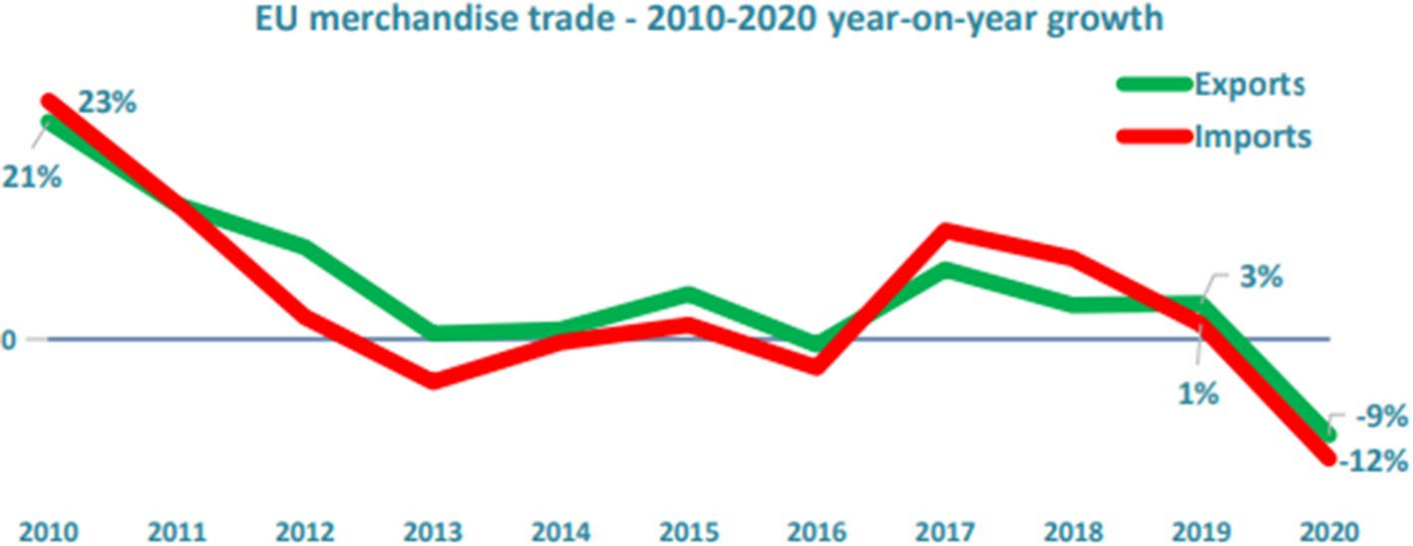
EU merchandise trade—2010–2020 year-on-year growth. *Source* (DG Trade 2021)

Due to lockdowns in Europe and the rest of the globe, the COVID-19 pandemic significantly impacted the European Union (EU) industry. Multiple supply networks across a number of industries experienced disruptions, most notably during the crisis's early stages and in the case of internationalised value chains. Unprecedented policy measures have been launched across Europe and the rest of the world to alleviate the effects of economic shock and aid recovery (De Vet et al. 2021). Brexit has resulted in a significant decrease in goods and services trade between the UK and the EU27. The two major influences on the cost of goods are the effect of pricing and the effect on the country's GDP (Felbermayr et al. 2017). As a result of these disruptions, trade flow decreased, as seen in Fig. 2.

The RoRo segment is seen to possess growth potential across the EU and is occasionally maintained with the assistance of EU funding. Since trailers compete with European shortsea container shipping, markets are expected to expand slower where fixed links are in use. However, if economies are not comparable in development or where links connect to less developed economies within (or beyond) the EU, RoRo cargoes may outperform.

There are around 40 RoRo/RoPax companies in northern Europe, according to Harbours Review (2017). Most businesses have specific geographic areas of operation and corresponding ports of operation, a few of them, for example, Stena Line, DFDS, and Cobelfret, render service to the whole of the North Europe region. However, the most remarkable shift in traffic volume is triggered by the UK's exit from the EU, as 144 of the 1,382 ports having marine traffic in Europe are located in the United Kingdom (ISL 2018). Furthermore, the number of vehicles travelling from Europe returning empty is more incredible because of Brexit and Covid 19. As a result, freight exports' value decreased in 2021 (Morris 2021).

The European Commission suggested reviewing one of the EU's major transport corridors, the North Sea-Mediterranean Core Network Corridor (NSM Corridor), which formerly included the UK, as part of Europe's contingency measures for Brexit. In addition, the European Commission has proposed new direct maritime routes connecting Dublin and Cork in Ireland with Zeebrugge and Antwerp in Belgium, as well as with Rotterdam in the Netherlands, in order to avoid the possible detrimental impact of UK customs inspections on Irish exports. These ideas have been augmented by critical recent developments in the sector, with key companies, such as Irish Ferries, Brittany Ferries, and CLdN, introducing extra capacity on continental routes by way of the MV Celine and the MV Laureline vessels, which both started operating in 2018 and 2019 respectively (Vega et al. 2021).

### Ireland freight transport

RoRo traffic in Ireland is based on the Northern, Central, Southern, and Continental maritime corridors. The Northern Corridor uses Northern Ireland's relatively short transit times with Scotland. The Central Corridor is served by vessels calling at Holyhead, Liverpool, and Heysham from Dublin, while the Southern Corridor is served by RoPax vessels calling at Fishguard and Pembroke in Wales. Alternatively, the direct continental Corridor, which previously consisted of RoPax vessels connecting the ports of Rosslare, Dublin, and Cork to the ports of Cherbourg, Brittany, and Roscoff in Northern France, has undergone significant changes with the arrival of a new operator, Cobelfret, which began operating direct RORO services from Dublin to Zeebrugge and, recent times, Rotterdam (Vega et al. 2018).

The UK, a peripheral island country, is a significant trading partner for Ireland and acts as an essential transit network for Europe's market. Historically, Irish merchants have used the UK's transit system to move products between Ireland and continental Europe, thus utilising the UK as a bridge (Vega et al. 2018; Breen et al. 2015).

Due to the high percentage of RORO cargo sent between Ireland and the rest of Europe, Great Britain has traditionally been a significant contributor to this route. Cargo movements have depended on the UK landbridge network's competitive and efficient service, which is backed up by a high degree of dependability and security. According to the Irish Exporters Association, two-thirds of its members reach continental markets via the UK landbridge. According to the Economic and Social Research Institute (ESRI), the UK land bridge accounts for 53% of Irish exports to all nations except the UK (Vega et al. 2021).

Landbridge is beneficial for connectivity and competitiveness reasons. Ireland is a component of the North Sea Mediterranean Core Network Corridor, including the UK. The Corridor of the Core Network identifies the most critical links within the EU's Trans-European Network (TEN-T). The TEN-T initiative is aimed at establishing and expanding a pan-European network of roads, railway lines, inland waterways, marine shipping routes, ports, airports, and rail-road terminals. Brexit substantially alters the Corridor's design and operation, with implications (Breen et al. 2015).

For example, it is projected that 26,000 tonnes of fish were transported via the UK's landbridge in 2017 to reach its markets. The annual number of fresh fish carried over the landbridge is about 1300 trucks. While transport of fresh fisheries products accounts for a relatively minor part of total RoRo traffic, the EU continental market is a critical market for those goods, and the landbridge route to that market accounts for a significant amount of that trade. An increase in transit time has an adverse effect on shelf life in this sector, causing the industry to lose competitiveness.

According to Tetlow and Pope (2020), the structure of Ireland's freight transport supply to the continent, as well as the entire Irish haulage sector to be significantly affected by the move away of maritime freight routes from the UK land bridge. As shown in the figure below, Numerous ferry operators have had to cancel multiple transits due to traffic congestion because of various Covid requirements and Brexit-related alternatives.

**Fig. 3:**
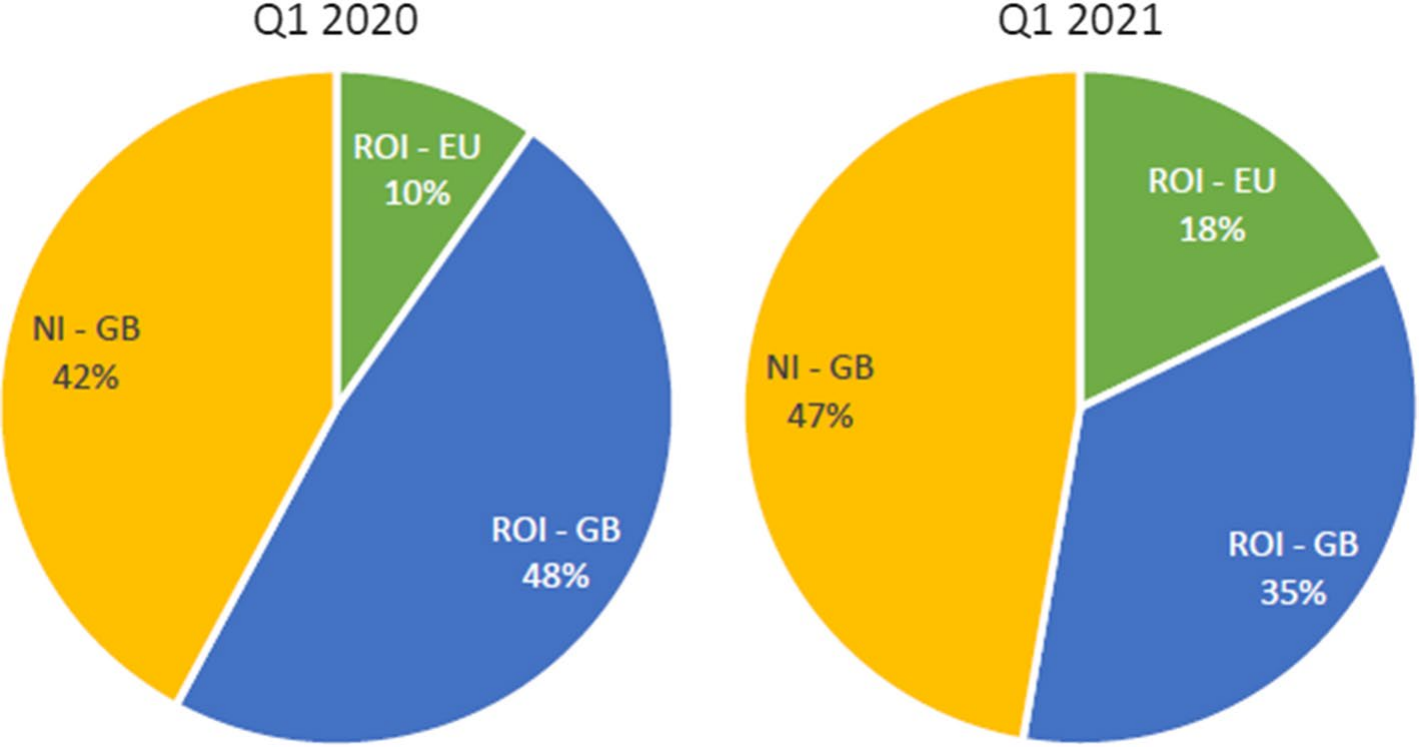
Shifting Traffic Patterns between Q1 2020 to Q1 2021. *Source* (IMDO 2021a)

According to Vega et al. (2021), Ireland's freight transport industry is transitioning from a primarily road-based system with minimal marine participation to a fully integrated multimodal maritime system. In addition, new measures are being explored to reduce Ireland's reliance on the UK land bridge in a post-Brexit scenario marked by considerable uncertainty regarding the EU's future relationship with the UK. Due to covid, restrictions in March and October 2020 decreased RoRo volumes in all ports. Although the 'first wave' decrease in March 2020 was substantially more severe in terms of volume, the impact on the shipping sector was immediate in both instances and impacted all segments of the market. Along with the restrictions, new customs regulatory requirements to trade between the Republic of Ireland and the United Kingdom resulted in a 47 per cent decline in value terms in EU imports from the United Kingdom in the first two months of 2021, as shown in Fig. 3.

According to IMDO (2021a), ROI records a traffic shift between the first quarters of 2020 and 2021 due to an exceptional shift in traffic caused by changes in EU-UK trading restrictions. There was a noticeable rise in traffic on direct ROI—EU services, but a noticeable fall in ROI—GB traffic. As a result of these adjustments, the ROI—EU raised its proportion of total island volumes from 10 to 18%, while the NI—GB increased its share from 42 to 47%. Importers and exporters who opted for direct EU services benefited from an exceptional increase in RoRo capacity added by shipping operators. Shippers responded by doubling available capacity on direct RoRo services to continental Europe in the RoRo sector. However, concerns over delays and inconvenience caused by additional customs procedures on transportation between the UK and the EU motivated the decision to abandon the Landbridge (IMDO, 2021c). The following section examines the ferry and port operators' industries.

## RoRo ferry operators and port analysis

Reliability, operational effectiveness, and the capacity to safely and efficiently carry freight have always been priorities from the beginning of the COVID-19 problem. Combined with the UK's withdrawal from the EU, it necessitated swift action by the major stakeholders in freight movements. Ports and RoRo ferry operators have had to adapt their operations and change their governance and communication procedures. In addition, they redoubled their efforts to collaborate with users and stakeholders (UNCTAD 2021).

As mentioned before, the lower trend in national GDP growth, as well as the lockdowns and/or restricted function imposed by Brexit and COVID-19, have been connected with less maritime commerce, interrupted cargo flows, and fewer ship calls, and declining connection levels. While ports and RoRo ferry operators seek ways to recoup tonnage handled before the epidemic, they are also looking for additional cargo flows and ways to offset the pandemic's financial repercussions. Shipping, ports, and supply chain players all exhibit varying capacities for adapting to each of these shocks. These capacities may represent the diversity of hazards encountered in each industry and the players' varying capacities to predict, plan for, respond to, and recover from severe multi-hazard threats influencing and disrupting marine networks. Such distinctions may arise due to disparate strategies, policies, and governance procedures or simply from divergent approaches to functional/operational characteristics.

### Port response strategies

Port operations were considered "critical services." As a result, businesses continued to operate despite national economies being shut down. Additionally, their employees were classified as essential workers, allowing them to engage in everyday port operations. A first operational modification was the priority of port operation in order to sustain the freight transport and logistics chains and assure the delivery of commodities required to combat the epidemic. Fast lanes were constructed for essentials such as medical cargo and foodstuffs for continuous movement. These priority lanes ensure the availability of pilots, tugboats, cargo handling services, and vehicles. Ports throughout the United Kingdom, Northern Ireland, Ireland, and Europe are listed in the table below (Table 3).

**Table 3:** Port across the United Kingdom, Northern Ireland, Ireland and Europe

*England including Wales and Scotland*		
Cheriton Channel Tunnel Terminal at Folkestone	Holyhead	Pembroke
Dover	Hull	Plymouth
Felixstowe	Immingham	Poole
Fishguard	Killingholme	Portsmouth
Harwich	Liverpool	Purfleet
Heysham	Newhaven	Sheerness Teesport
*Europe*		
Rotterdam	Palermo	Sagunto
Amsterdam	Savona	Tarragona
Antwerp	Salerno	Santander
Zeebrugge	Genova	Piraeus
Bremerhaven	Valencia	Limassol
Hamburg	Pasajes	Derince
Civitavecchia	Vigo	Gemlik
Livorno	Malaga	Izmir Martin
*Ireland*		
Bantry Bay Port Company	Dundalk Port Company	Port of Cork
Drogheda Port	Greenore Port	Port of Galway
Dublin Port Company	New Ross Port	Port Of Waterford
Dun Laoghaire Port Company	Shannon Foynes Port Company	Rosslare Europort
		Port of Cork Wicklow Harbour
*Northern Ireland*		
Belfast Harbour Company	Port of Larne	Warrenpoint Harbour Authority

*Source* Own

Ports enforced sanitary precautions following national and municipal authorities' rules and recommendations. These guidelines and recommendations were considered when developing internal and terminal operations procedures critical steps.

For example, Dover port-maintained operations to guarantee that vital products supplying supermarkets, the NHS, and companies throughout the country continued to flow via the ferry and cargo terminals. Simultaneously, intensive planning and preparation for the end of the Brexit transition period were undertaken in collaboration with the UK and other governments to ensure the port received enough assistance. As the primary UK gateway to the EU, the port planned a significant project to reconfigure the outbound controls to increase the capacity of the French passport control, reorder the controls to detect non-compliant traffic prior to the French border and adapt to future changes to the EU immigration system (Port of Dover 2020). Similarly, in June 2020, Peel Ports announced the addition of a new stop at the Port of Liverpool, launching the first pure RoRo service with CLdN on a triangle route connecting Santander (ES), Liverpool (UK), and Dublin (IE) (IE). The new route will run a weekly connection between Spain, the United Kingdom, and Ireland. The new RoRo route has been essential in protecting and preserving each link in the supply chain. In addition, it can reduce lengthy European transport legs to maintain a robust and dependable service in the face of COVID-19 limitations (Peel Port 2020). Antwerp reduced shift staffing levels by forming an additional standby team of workers.

Similarly, Hamburg decreased its shift count by establishing a standby crew. Marseilles decreased staff in traffic towers, even though the port's capacity was limited to a maximum of three simultaneous ship manoeuvres. To combat the spread of illness, Gothenburg implemented work rotation programmes in which individuals worked independently. This guaranteed that critical knowledge was always available and that the freight hub remained operating (UNCTAD, 2021). Additionally, Dublin, despite the uncertainty created on routes to Rotterdam, Santander, and Liverpool and the expansion of capacity on current Liverpool services (Dublin Port 2020).

Several ports and other supply chain players have estimated the possible financial effect of pandemic-related disruptions and developed scenarios for the ports' vulnerability to the impact of changing cargo volumes and revenues. Numerous ports have referred to financial issues. Most ports have seen minor revenue losses and a tolerable disturbance in operations. For completely privatised profit-oriented ports, the situation has been more difficult. Cabinet Office (2020) announced the UK government's Port Infrastructure fund using British ports as an example. This fund was established to enable maritime ports and other sectors that handle goods imported from the EU to access funding for the construction of necessary infrastructure and facilities to enable customs and sanitary/phytosanitary checks to be conducted at ports following the transition period's conclusion. This will entail expanding and improving the current infrastructure. The fund will assist the development of infrastructure and facilities that will improve the flow of EU trade into the UK. However, the DfT (2020) announced that 41 of the 53 ports applied were successful, and a total of £200 million has been temporarily granted. Twelve ports were deemed ineligible or were rejected during the assessment process. Nonetheless, data collected by the British Ports Association (BPA) indicated that 55% of the UK's ports were dissatisfied with the existing public mechanisms and funding available to British businesses to address pandemic-related challenges and called for the establishment of Government COVID-19 debt underwriting schemes. Other ports have achieved financial stability by, among other things, putting new contracts on hold and negotiating with suppliers to prolong payment periods in order to reserve funds (BPA 2020).

According to UNCTAD (2021), some minor delays were unavoidable as activities resumed and continued to adapt in various areas of the world beginning in the second quarter of 2020. These include delays connected with loading and unloading unaccompanied trailers on cargo Ro/Ro ships, as well as delays associated with loading and unloading automobiles on car carriers as a result of worker distancing measures. For example, the Port of Dover suspended traffic from the UK owing to French border restrictions.

In the late twentieth and early twenty-first centuries, road haulage services, along with RoRo and container shipping, enabled the development of just-in-time supply chains and lean manufacturing, where, for example, automotive components can be imported from the rest of the EU or the rest of the world for assembly in a car plant in the UK (MDST 2019). Accompanied RoRo freight entails a commercial haulier transporting products to a departure port via vehicle, boarding the vessel to the destination port, and then transporting the goods to their final destination. Unaccompanied RoRo freight entails a haulier transporting products to a ferry port and then leaving them with the ferry operator to be transported to the destination port, where they are retrieved and delivered to their final destination by a different haulier (IMDO 2019). However, due to Brexit, ports are experiencing a driver scarcity to move trucks on roll on roll off.

The figure above illustrates how Brexit and the Covid 19 pandemic have contributed to the driver shortage. Many drivers returned to their country of origin due to Covid during lengthy periods of lockdown and limited movement. However, the overwhelming majority have yet to return. Additionally, the uncertainties surrounding Brexit and future rights to live and work in the UK pushed many drivers to choose the same course of action. Again, the overwhelming majority have not returned and are not anticipated to do so. Additionally, RHA claimed problems in training new drivers due to government limitations.

On the other hand, ports such as Peel Port can manage the shortfall by utilising the unaccompanied RoRo freight concept. This enables the management of available drivers, protects drivers from dangers, and provides authorities more time to perform border inspections.

### Ro-Ro ferry operators

The global economy began to decline progressively in the second half of 2018. As economic indicators continue to worsen, executives fear that momentum could be lost further in 2019 and 2020. GDP growth rates in the United Kingdom, Republic of Ireland, the European Union, and the rest of the globe have slowed, and the global RoRo industry's fulcrum has shifted (DFDS 2019). Since the United Kingdom's exit from the European Union (EU) or Brexit, there has been worry over the potential loss of maritime connection between Ireland and continental Europe (Vega et al. 2021). Major corporations often run ferry services. These firms compete by offering similar routes or lines to similar locations. Table 4 below lists RoRo Ferry companies are operating in the United Kingdom, Ireland, Northern Europe, the Mediterranean, and the Baltic Sea.

**Table 4:** RoRo Ferry companies across the United Kingdom, Republic of Ireland, Northern Europe, Mediterranean and Baltic Seas

North Europe include the United Kingdom and the Republic of Ireland	Mediterranean Sea	Baltic Sea
Ans Black Sea Ferry Clyde CondorFerries DFDS Seaways Euro Marine Logistics (EML) Finnlines Irish Ferries KESS Lillgaard Mann Lines NorthLink Ferries P&O Ferries SCA Logistics Sea-Cargo SeaTruck Ferries SOL Continent Line Stena Line Tallink/Silja Transfennica UECC Universal Logistic System UP Wagenborg Shipping Sweden	CMA-CGM CTN Ferries DFDS Seaways Ekol Euro Marine Logistics (EML) Grimaldi Lines Med Cross Lines Trasmediterranea Acciona UECC UN RO-RO	Akdeniz Ro-Ro

*Source* Author findings from Habour review 2017

As businesses prepare for Brexit, ferry companies such as Stena line and struck have boosted their capacity. Similarly, certain operators, such as the owner of Irish Ships, obtained financing from the European Investment Bank (EIB) to purchase new ferries to operate on the Dublin-Holyhead and Cherbourg routes. Additionally, P&O Ferries stated that it will re-flag its ships that sail in the English Channel to Cyprus (IMDO 2019).

After the transition period expired on December 31 2020, the pattern of RoRo freight travels altered due to new customs procedures for goods leaving or entering the UK. As a result, key supply chain businesses, such as the RoRo firms, experienced supply chain interruption. In addition, certain commodities, for example, such as ready meals, processed meat, and fruit were impacted owing to their short shelf life. As a result, store shelves are empty.

From January 1 2021, goods entering Northern Ireland from the United Kingdom was subject to inspection. Additionally, there have been delays at the Port of Dover. Asda, Sainsbury's, and Tesco informed BBC News Northern Ireland that certain items had been delayed. The transportation of fresh food was of concern to Hauliers (Andrews 2021). According to ICG (2021), traffic to and from the United Kingdom has been diverted from ports in the Republic of Ireland to ports in Northern Ireland, where customs checks have been suspended, or, in the case of landbridge movements to Continental Europe via the United Kingdom, to direct services between Ireland and France, where free movement of goods is permitted. The initial magnitude of this route shift is gradually reversing as clients become used to the increased custom procedures. However, the resumption of existing route-sharing arrangements between routes servicing Britain from Northern Ireland and the Republic of Ireland would be postponed as long as Northern Ireland's customs procedures are suspended beyond the transition time originally agreed between the UK and the EU.

The expenditures incurred by the ferry operation are critical since they impact the firm's financial success (Urbanyi 2015). Butt (2021) observed that COVID-19's influence is causing a ripple effect in supply chains that affects several industries. Additionally, COVID-19 causes both supply and demand fluctuations, both harmful. Investigating several facets of the RoRo ferry industry reveals that a single interruption can affect the operation of other supply chain functions (upstream or downstream). The quarterly report demonstrates that, depending on the type of occurrence, there is either an upstream or downstream effect.

As seen in the table above, most companies experienced a significant decline in 2020 due to Brexit and Covid. Numerous CEOs refer to 2020 as the 'hard seas' time. However, not all RoRo ferry companies report their revenue publicly. As a result, this table contains just a few RoRo companies. The co-existence of Brexit and Covid-19 issues have exposed truckers and haulage firms to supply chain risks on a global scale. As a result, many cargo owners and carriers have re-evaluated their transportation plans, opting for alternative ports, shipping methods, and modes of transport in order to maintain supply chains. Given the present limits on international travel, tighter border controls, and COVID-19 concerns, one of the most significant benefits of driverless methods is that the danger of delays connected with those specific issues is decreased.

Regarding capacity, ferries and RoRo vessels are sized using the maximum possible lane metres method. A lane metre is a stretch of a deck that is one lane wide and one metre long. As liner ships, Ro-Ros rarely have a fixed and precise maximum capacity. The cargo mix governs the capacity of RoRos and ferries in relation to moveable decks, internal ramps, lane heights, and other factors that may restrict the quantity of cargo that a vessel can carry. The global increase in Ro-Ro traffic necessitates capacity expansion at Ro-Ro ports. Because Ro-Ro shipments cannot be stacked, appropriate terminal capacity in Ro-Ro terminals is required (Özkan et al. 2016).

Due to the expected delays on the UK Landbridge due to Brexit and the implementation of Covid, maritime firms responded unprecedentedly, creating new options for direct EU RoRo services. Irish Maritime Development Office (IMDO) (2021b), states that shipping companies (Stena Line, Irish Ferries, CLdN, and Brittany Ferries) have increased capacity on direct routes until 2021. Among the destinations were Zeebrugge, Cherbourg, Bilbao, and Santander. In addition, DFDS, a new operator, began operating a RoRo service between Rosslare Europort and Dunkirk in January 2021. These measures added up to more than double the capacity for direct RoRo flights to continental Europe. The number of possible routes has risen from five to 134. Irish importers and exporters benefited from a considerable expansion of direct EU services available in 2021. Following a rise in 'direct demand,' Irish merchants now have access to 12 specific direct EU RoRo services, up from five in 2019 (Bailey and Treacy 2021).

Additionally, Multimodal UK adds that in the post-Brexit environment, CLdN is increasing shipping capacity with the inclusion of extra sailings on its UK and Irish routes, providing clients with 12 weekly sailings. Irish ferries increased capacity on the Dover—Calais route, aiming to considerably enhance the landbridge's capacity and dependability for exporters and importers. On the Dublin—Holyhead, Rosslare—Pembroke, and Dover—Calais routes, hauliers now have a single operator providing an all-inclusive service. This will facilitate exporters' and importers' access to European markets under the Common Transit Convention. Additionally, DFDS begins service between Sheerness and Calais. The new route complements the current network of flights connecting the United Kingdom to Europe and responds to the rising demand for unaccompanied freight services (Multimodal UK 2021).

## Research design and approach

The design for this project considered the nature, strategies, and methodological choices leading to the acquired potential ethical concerns for the research project. Ontology and interpretivism epistemology is considered as the nature of this research as the aim to analyse a RoRo ferry industry amidst different concerns requires knowing the realities (the past, ongoing and assumption of what is to happen) in the industry researched.

Ontology is the assumption about the nature of reality (Saunders et al. 2016). Several assumptions have been made pre-Brexit and the occurrence of covid 19 has further brought about various assumptions about their effects to the RoRo ferry industry. For instance, Vega et al. (2018) opines in their research that the impacts from post-Brexit and Covid could lead to a record of modal and route shift such as the RoRo traffic on the UK landbridge will shift to transport through diverse modes and shift from road-based freight transport to more economical transport modes such as Short Sea Shipping (SSS). Likewise, Tetlow and Pope (2020) further opine that the industry will undergo delay due to a lack of domestic workers as a result of the pandemic. Also, leaving many ferry operators to cancel several transits due to traffic as a result of various Covid requirements and alternatives in place after Brexit. It is of importance to take this assumption and base them on realities which further draws the nature of this research to be interpretivist epistemology.

From the meaning of epistemology which constitutes the assumptions about knowledge and how it can be communicated to others. This research shows its acceptable knowledge ranging from numerical data to textual and visual data, from facts to opinions, and including narratives and stories. The research paradigm shows an interpretive epistemology as it interprets the new knowledge from the realities of the occurring events which is the Brexit and Covid 19, its effects and its resilience by the RoRo ferry industry in regards to supply chain and its geography and political economy. This research is more of a description-explanatory study with a mix of evaluative research likewise, deductive research approaches for data analysis. Using a method mapping gave a clear research paradigm used for this research project (O'Gorman 2015). Being a description-explanatory research, the project was able to ask questions that start with what, how, and when so as to gain a broad description of the event and further able to study the problem of an event in order to explain the relationship between variables.

Likewise, the research was considered to be exploratory and evaluative as the latter allows findings on how effective the different strategies implemented by the organisation are utilised, why and then comparing the explanation to existing theories, and the former allows to explore the event history. A deductive approach is used to evaluate hypotheses associated to existing theories. The approach allowed explanation between the concepts and variables (Saunders et al. 2016). Considering the form of mixed research philosophy and approach, it shows the need of multiple methods of research. The mixed method design approach as a branch of multiple methods research was used for this research, which entails the use of both quantitative and qualitative research approaches. A convergent parallel design is a set of observable results obtained from both quantitative and qualitative data that has been collected and analysed (Creswell 2018). As a result, this study employs a convergent design. This design was chosen to compare or relate the outcomes of both qualitative and quantitative data, leading to a greater knowledge of the research project.

## Data collection and limitations

The choice of data gathering methodologies is linked to the research's overarching theoretical and epistemological position. This procedure entails gathering relevant information from reputable sources about the area of study solely for the goal of achieving the research aim and objectives (Oliver 2014). This project employs primary and secondary data gathering techniques. The secondary data collection involves data from other sources by other researchers. This project used quantitatively and qualitatively data from sources such as journal articles, books, publicly available reports, and databases such as ferry traffic database and revenues, publicly available surveys, documents and company report. With the use of primary data for collection, this data are original generated data based on the purpose to be achieved for the research. The primary comprises two data techniques, namely qualitative and quantitative data. Qualitatively and quantitatively, this research gathered data through a web-based survey for the latter and use of observations from secondary sources for the former to understand a broad view of the research (identifying the problem, the strength and ongoing challenges) from selected groups.

The research analysis experienced limitation due to small sample data. Eighty (80) were distributed to sample group due to their specialisation as key actors in RoRo freight movement. Fourteen (14) responses were received. Due to this reason, there was delay in continuation of research, as calls had to be put through to each sample groups. Nevertheless, only very few responded to answering the survey. As a result of small sample size, only basic statistical analysis was carried out and it limits the statistical power of understanding more relationship between variables.

The questionnaires were distributed to respondents electronically using Google forms, and respondents either accessed the questionnaire through a web browser using a hyperlink (Web questionnaire) on their computer, tablet or phone, which was shared with the respondent through emails, social and professional platforms such as LinkedIn, WhatsApp and Facebook. For accuracy, various factors such as selecting a group of respondents, ways of reaching out to respondents, the format in which questions are being asked, the number of questions and the size of sample required for analysis are considered. A selected group of experts, namely RoRo ferry operators, logistic company managers, RoRo ferry users, port operators, policymakers and haulier operators, were the target as respondents due to their knowledge and role in the field explored. In addition, the validity and reliability are tested, ensuring questionnaires measure the study intended and the data collected correlates.

The questionnaire comprised of both closed and open-ended type questions. Both question types were used instead of one as the closed question type allowed two or more alternative answers for the respondent, and the open type gave the respondent the room to respond in their own way to know their opinions. To ensure that respondents will not have problems understanding and answering the questionnaires, the review was carried out by expert supervision. After the final check, questionnaires were sent to the target groups.

## Data analysis

According to Castellan (2010), this procedure entails carefully assessing and selecting important information from data acquired from many sources in order to establish a structure for analysing the study work. Saunders et al. (2016) suggest that data analysis is the most significant element of a research project since it helps simplify the complexity of data collected into concise information that produces outcomes and provides answers to research questions. The research used a deductive approach using descriptive and inferential statistics to analyse the data collected from a mixed data collection technique. Likewise used is an inductive approach to analyse the qualitative data collected through existing secondary theories. Descriptive statistics helps to describe the data analysed, while Inferential statistics allow for the creation of predictions based on descriptive statistics. To make inferences about a population or firm starts with a hypothesis. Brown and Saunders (2008) pointed out that a hypothesis is a statement or assumption about the relationship between two or more variables that are tested to see if the evidence supports that hypothesis.

## Analysis approach

Using a deductive approach for quantitative data collection, a survey was carried out using questionnaires. Surveys, according to Sapsford (2007), entail systematic observation. They pose the questions that the researcher wants to answer, and they frequently specify the range of possible responses. The goal of survey research is to get consistent replies to consistent questions; hence standardisation is essential. Every respondent was asked the same exact questions to be answered. Quantitatively, data analysis was carried out using descriptive statistics and inferential statistics. The questionnaire was first coded having in mind categories to be analysed and transferred to a statistical software package. Coding is the process of giving numbers or other characters to data responses received to classify or categorise them (Brown and Saunders 2008). A statistical software package termed IBM Statistical Package for Social Sciences was used for analysis. The data collected are organised in a certain way so that the statistical package can read them. The coded data was transferred to the software and descriptive analysis was acquired. The descriptive analysis.

The research took a deductive approach, analysing the data obtained using a mixed data collecting strategy with descriptive and inferential statistics. First, the questionnaire data are analysed using a specific statistical package called the IBM Statistical Package for Social Sciences (SPSS). Then, the data collected from each respondent was coded, variable names were assigned, and the data was input into SPSS for analysis. Similarly, inductive analysis was utilised to analyse the qualitative data gathered.

The section below contains descriptive statistics that illustrate the data's facts.

## Descriptive statistics

Questionnaires were distributed to respondents to analyse data. Questions were created in response to the study topic of understanding the difficulties connected with RoRo ferry industry-level indicators due to the impact of Brexit and Covid 19 between the UK, Ireland, and continental Europe. A clear understanding allows the research to achieve its aim and arrive at a conclusion.

Table 5 below shows the frequency, valid percent, and cumulative percent of the response to the respondent's organisation. There are no missing data in this question. A total of 14 respondents from the UK and the EU answered the question "Which of the following best describe your organisation?". Three (3) respondents answered to be ro-ro ferry operators, five (5) hauler operators, one (1) respondents from a logistic company, two (2) port operators, and three (3) respondents from other organisations, namely Customs Broker, Irish Exporters Association and MET. As different players are involved in freight movement, and all connected to ro-ro freight movement, the researcher asked this question to understand everyone’s perspective by their organisation.

**Table 5:** Respondent Organization by Target grouping

Which of the following best describes your organisation?			
	Frequency	Valid percent	Cumulative percent
*Valid*			
RoRo ferry operator	3	21.4	21.4
Haulier operator	5	35.7	57.1
Logistic company	1	7.1	64.3
Port operator	2	14.3	78.6
Other	3	21.4	100.0
Total	14	100.0	

*Source* Own

**Fig. 4:**
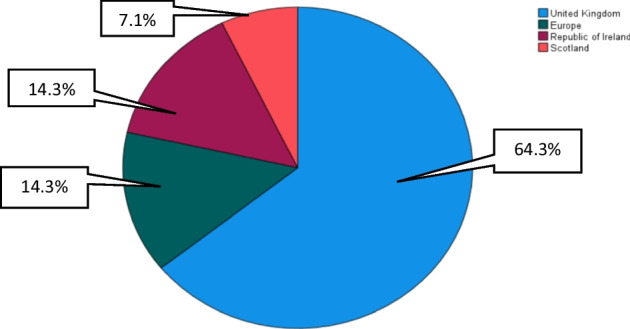
What country of residence is your organization?. *Source* Own

Transportation of freight by RoRo ferry is commonly used within the countries of study. These countries share maritime links during transportation. According to Anca (2019), collaboration and coordination across countries are necessary for an efficient supply chain. This question of country of residence allows the researcher to ascertain the location of the respondent organisation to base realities on existing theories. Figure 4 shows that 64.3% of the respondent answered the United Kingdom, 14.3% Europe, 14.3% Republic of Ireland, and 7.1% respondent organisation is in Scotland.

**Fig. 5:**
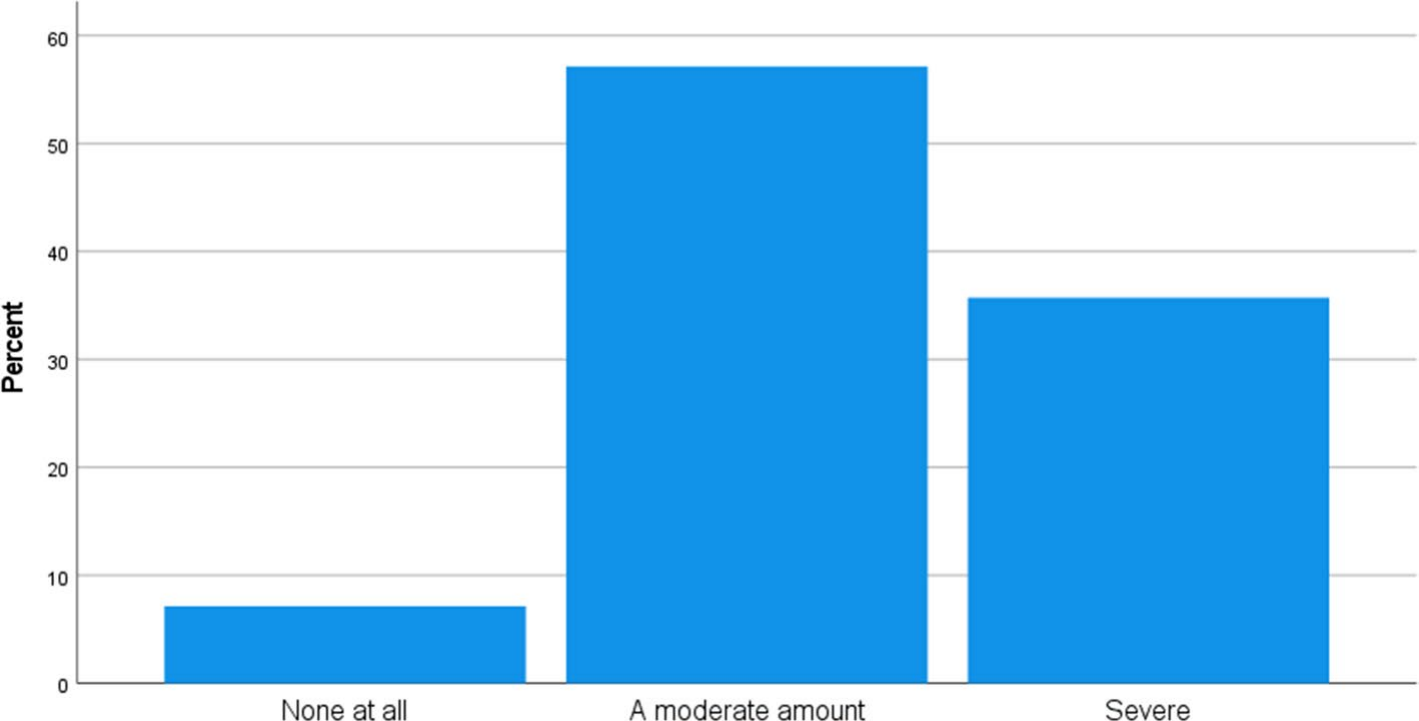
How Brexit and Covid being of impact in your organisation?. *Source* Own

The co-existence of Brexit and Covid 19 has had of negative impact on organisations. However, the impact on organisational performance differs from another. In responding to the question "How has Brexit and Covid been of impact in your organisation?" reveals the impact on each respondent organisation. Figure 5 shows that 57.1% had a moderate impact, 35.7% had a severe impact, and 7.1% recorded no impact on the organisation. As a result, most firms were negatively impacted by Brexit and Covid 19, ranging from moderate to severe.

**Fig. 6:**
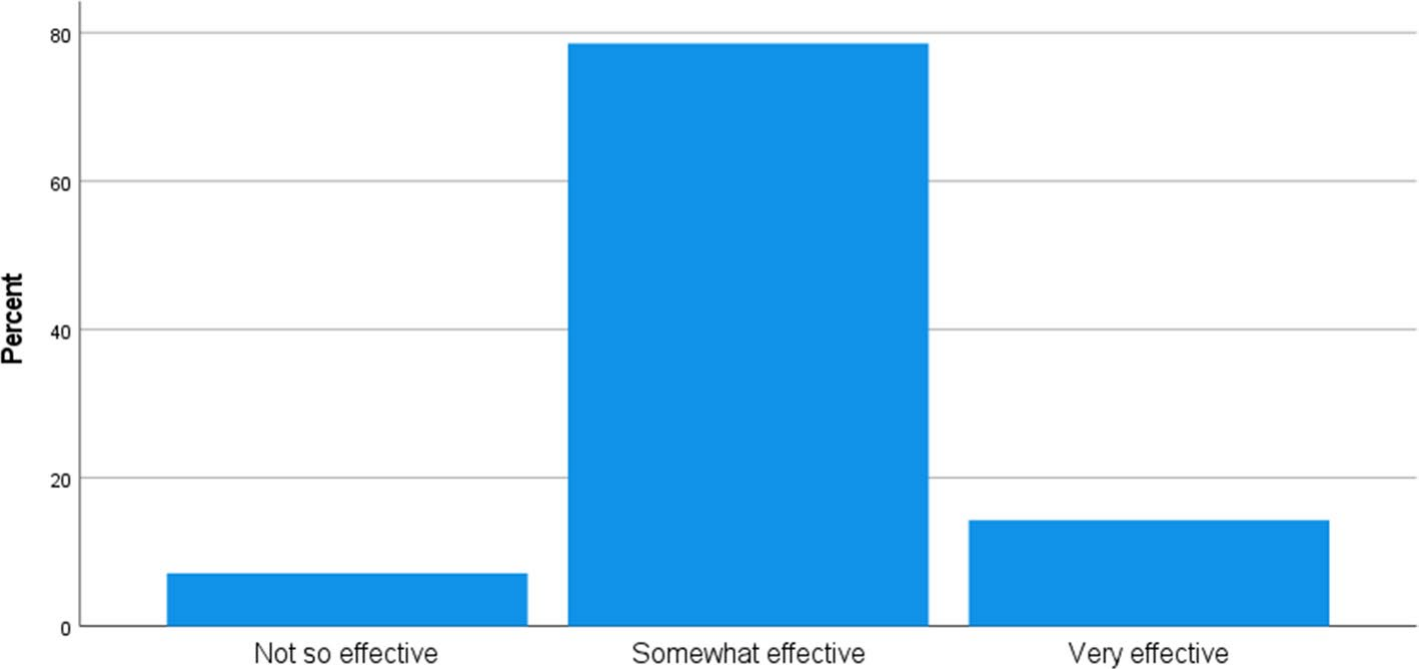
Have the political initiatives and interventions to address Brexit and Covid to organisation been effective so far?. *Source* Author

According to Avelar-Sosa (2018), the government at all levels is responsible for enabling business activity by simplifying bureaucratic procedures, providing services such as phone, internet, land, facilities, and taxes, among other things. In other words, the government provides the infrastructure that permits raw materials and completed goods to flow. The "Have the political initiatives and interventions to address Brexit and Covid to your organisation been effective so far?" question is intended to determine the impact of political intervention and activities on respondent organisations in relation to Brexit and Covid 19. Figure 6 shows that 78.6% consider the initiatives put in place by the government to be somewhat effective, 14.3% to be very effective, and 7.1% consider as not so effective.

Respondents we asked to state three major areas Brexit has been of Negative affect their organisation in order to understand areas in which Brexit has been of effect to the respondent organisation. Table 6 shows the exact responses from the respondents. Acquired frequent themes from these responses are extra cost, more paperwork, traffic delay, time loss, border delays, driver shortages, extra training cost, trade division, export reduction, increased taxation, decrease in freight volume, shortage of workers, and extra workload.

**Table 6:** Major areas Brexit has been of Negative effect in the respondents'' organisation

	Frequency	Valid percent	Cumulative percent
Valid	1	7.1	7.1
Additional costs to create customs paperwork, delays at customers and/or ports to enable generation of paperwork, significant amount of uncertainty and training associated with the additional requirements following Brexit	1	7.1	14.3
Border delays, Driver shortages, Increased admin time- more paperwork	1	7.1	21.4
Decreased UK exports, created a divide for UK & EU traders	1	7.1	28.6
Increase in taxation, Bilateral relations with other neighbouring countries	1	7.1	35.7
Indirect impact from Unavailability of haulage drivers	1	7.1	42.9
Mobilities, training	1	7.1	50.0
Port increased operative measures	1	7.1	57.1
Port operative process, Trailers been stuck, UK borders restriction	1	7.1	64.3
Reduce freight volume	1	7.1	71.4
Rising costs. Business relationships. Product or service sourcing and hiring staff	1	7.1	78.6
Shortage of drivers, restrictions from border checks	1	7.1	85.7
Shortage of drivers, shortage of workers, restrictions due to crossing border policies	1	7.1	92.9
Their understanding and implications of Brexit border controls have generated a considerable workload. Also, the very considerable developments in supply chains following the break. Work in advising manufacturers of alternative sources of materials and components	1	7.1	100.0
Total	14	100.0	

Another question investigated three major areas Covid has been of negative effect to the respondent's organisation and has been posed as an open typed question asked to understand areas in which Covid has been of effect. Table 7 shows the exact responses from the respondents. Acquired frequent themes from these responses are difficulties in communication, shortages of products, driver shortage, delay in traffic, shortage of labor, laying off workers, slow decision-making process, low profits, mobilities and training, temporary closings, and financial fragility.

**Table 7:** Three major areas Covid has been of negative effect to the respodents' organisation

	Frequency	Valid Percent	Cumulative Percent
Valid	2	14.3	14.3
Covid and home working has made interaction between personnel more difficult. Covid effects on supply chains have been massive and this, along with solutions, must be fed to members. Shortages of products	1	7.1	21.4
Driver Shortage, Delay in traffic	1	7.1	28.6
Impact on labour. Not of high impact on RoRo freight but more impact in regards to passengers	1	7.1	35.7
Laying off workers, Slow decision-making process, Low profits	1	7.1	42.9
Loss of labour (temporary) with associated loss of capacity for customer deliveries, additional risks to employees (many of whom are older due to the nature of the industry) from COVID due to spending time on RoRo vessels leading to the apprehension of employees and lower levels of confidence/morale, an additional requirement for testing which takes place in-house leading to additional risks for office-based employees and additional administration	1	7.1	50.0
Mobilities and training	1	7.1	57.1
Shortage of drivers	1	7.1	64.3
Shortage of labour	1	7.1	71.4
Shortage of labours due to isolation and most staff working from home	1	7.1	78.6
Shortage of staff due to isolation	1	7.1	85.7
Temporary Closings. Financial Fragility -Seeking of loans and grants. Shortage of staff due to isolation	1	7.1	92.9
Uncertainty in every aspect of business, ability to plan for business, staff absence	1	7.1	100.0
Total	14	100.0	

**Fig. 7:**
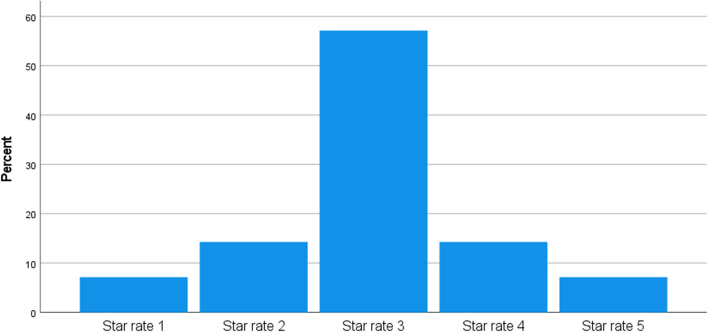
Rate UK/EU Government understanding of the problem that Brexit and Covid has caused on continuous ro-ro freight movement?. *Source* Author

Government understanding of the problems caused by these disruptions will allow necessary initiatives to be put in place. According to Sys and Vanelslander (2021), the link between countries means that one country's decisions might positively or negatively impact the other. The question “Rate UK/EU Government understanding of the problems that Brexit and Covid have caused on continuous ro-ro freight movement?” shows the respondent's reflection of UK/EU government understanding. 57.1% of respondents opine star rate three, 14.3% star rate four,14.3% star rate two, 7.1% star rate one and 7.1% star rate five showing that different organisation has different opinions of UK/EU government understanding (Fig. 7).

Dealing with either of these shocks has been difficult for sectors which include RoRo freight firms' organisations. However, its impact and management of it vary from one organisation to another (Tetlow and Pope 2020). The question "How has your organisation managed with the disruption due to Brexit and Covid 19 pandemic?" reveals how the organisation has managed despite the Brexit and Covid 19 pandemic. Table 8 below shows that 50% met their expectations, 42.9% exceeded expectations, and 7.1% managed below expectations. This shows that most organisation has good resilience to this disruption.

**Table 8:** Response to organisational management despite Brexit and Covid 19

How has your organisation managed the disruption due to Brexit and Covid 19 pandemic?			
	Frequency	Valid Percent	Cumulative Percent
Below expectations	1	7.1	7.1
Met expectations	7	50.0	57.1
Exceeded expectations	6	42.9	100.0
Total	14	100.0	

*Source* Own

**Fig. 8:**
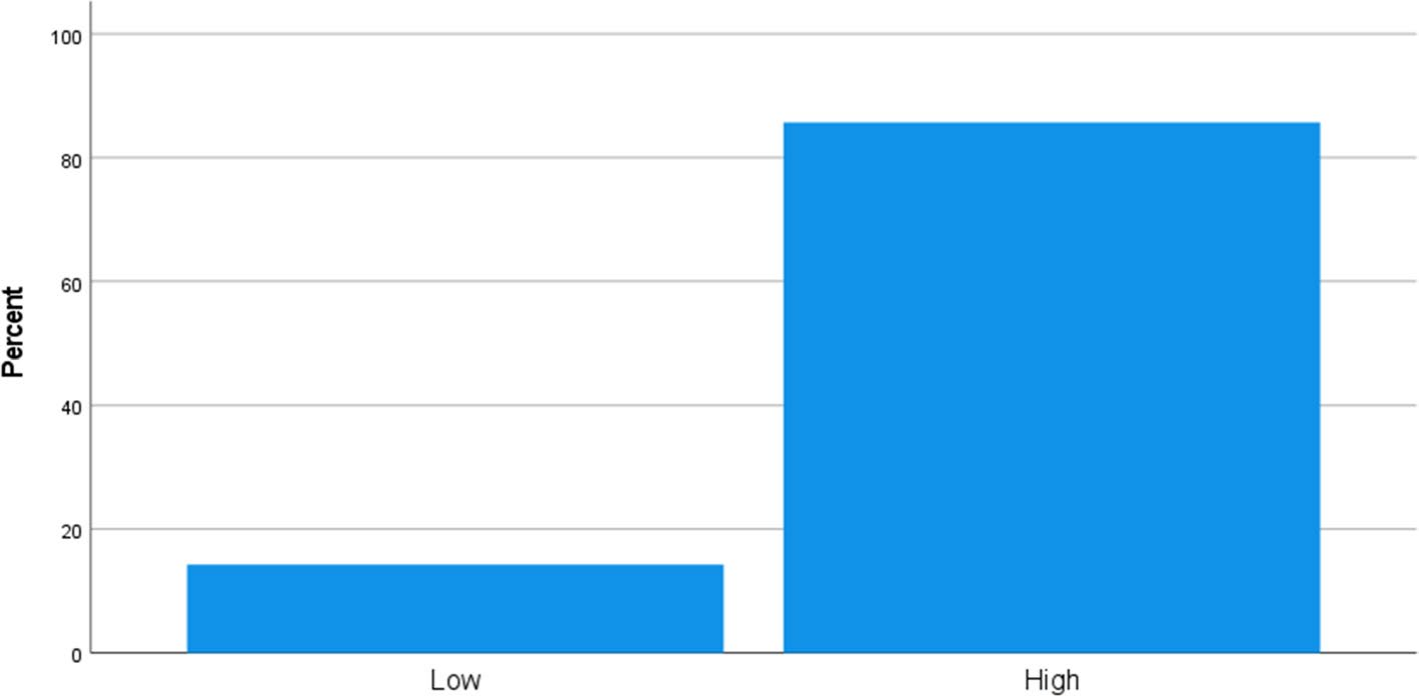
Based on the previous question, choose either High or Low to describe your organization survivability. *Source* Author

Organisations, when faced with a crisis such as Brexit and Covid 19, may disrupt business survivability. As a result, from the question "Based on the previous question, choose either High or Low to describe your organisation's survivability.", organisation management from short to long term reveals its survivability within a period. Despite the difficulties, 85.7 per cent of responders have a high survivability rate, as seen in the graph below. While 14.3 per cent suggests low survivability, it shows that some businesses are still fighting to recover from the effects of Brexit and the Covid 19 epidemic (Fig. 8).

This question, "Mention three strategies your organisation has implemented for continuous freight movement despite the Brexit and Covid 19 pandemic." is an open typed question asked to understand urgent strategies by RoRo freight actors for continuous freight movement. Table 9 below shows the exact responses from the respondents. Acquired frequent themes from these responses are additional capacity, unaccompanied freight mode, alternative routes, direct routes, hygiene measures, customs brokerage, increased cost, action call, terminal expansion, flexible crew, staff furlough, staff training, and effective communication.

**Table 9:** Strategies implemented by respondent's organisation for continuous freight movement despite the Brexit and Covid 19 pandemic

	Frequency	Valid Percent	Cumulative Percent
	3	21.4	21.4
Additional capacity-pure RoRo, following hygiene measures, unaccompanied freight mode	1	7.1	28.6
Alternative transport movement	1	7.1	35.7
Continuous effective operation	1	7.1	42.9
Created a customs brokerage to facilitate freight customs documents	1	7.1	50.0
Increase in haulage cost, Alternative routes, Move of operations to the EU	1	7.1	57.1
Letter call for action to government	1	7.1	64.3
More unaccompanied RoRo freight movement, increased routes, terminal expansion	1	7.1	71.4
Preparation in advance, capacity increase, cost-effective alternative to the traditional route	1	7.1	78.6
Significant research into the requirements (the information available from Governments is not clear), training of office-based and driving staff, and establishing accounts with all available ferry operators as a backup in the event of a delay	1	7.1	85.7
Using flexible capacity, Flexible crew, staff put on furlough instead of redundancy	1	7.1	92.9
Various strategies includes the following:	1	7.1	100.0
Valid Total	14	100.0	

This question "According to year revenue, select (Increase or decrease) your organisation's revenue growth in comparison to the previous year from 2016 to 2021, e.g., 2017-Increase, 2018-Increase, 2019-decrease, 2020-decrease, 2021-Increase" presents the revenue growth of each respondent organisation. For example, Table 10 shows below a collection of results from 2017 to 2021 that 78.6% increase in revenue growth and 21.4% indicated decrease. This agrees with the survivability response of the organisation, even if there has been a decrease in some years but not a high difference.

**Table 10:** Revenue Growth

Revenue growth frequencies			
	Responses		Percent of
	N	Percent	Cases
*Yearly Increase and decrease*			
decrease	15	21.4%	107.1%
increase	55	78.6%	392.9%
Total	70	100.0%	500.0%

*Source* Author

### Inferential statistics

This section presents a testable statement termed hypothesis to speculate the relationship and difference between variables.

### Correlation

Correlation analysis shows us how closely variables (both dependent and independent) are connected (Wagner III 2009).

#### Correlation analysis 1

The correlation table below tests the relationship between the UK/EU Government's understanding of the problems that Brexit and Covid-19 pose to continuous ro-ro freight movement and the impact of Brexit and Covid-19 on organisations operating ro-ro freights. The result shows the relationship between the two variables to have a high negative correlation and is statistically significant (r = − 0.799, *p* < 0.01). This inverse correlation indicates that an increase in the government's understanding of the problems associated with Brexit and the Covid-19 pandemic could lower the impact of these two events on organisations in the ro-ro freight business. Inversely, the failure of the government to understand the challenges Brexit and Covid-19 pose to continuous ro-ro freight movement may increase the likelihood of organisations being adversely impacted (Table 11).

**Table 11:** Correlation Analysis 1

		Rate UK/EU Government understanding of the problems that Brexit and Covid-19 have caused on continuous ro-ro freight movement?	How have Brexit and Covid-19 been of impact on your organisation?
Rate UK/EU Government understanding of the problems that Brexit and Covid-19 have caused on continuous ro-ro freight movement?	Pearson Correlation	1	
	Sig. (2-tailed)		
How have Brexit and Covid-19 been of impact on your organisation?	Pearson Correlation	− .799^**^	1
	Sig. (2-tailed)	.001	

*Source* Own

^**^Correlation is significant at the 0.01 level (2-tailed)

#### Correlation analysis 2

The relationship between an organisation's ability to manage the disruptions caused by Brexit and Covid-19 pandemic and the organisation's survivability was also tested. The result in Table 12 shows the relationship to be moderately positive and statistically significant (r = − 0.573, *p* < 0.05). This implies that companies who can effectively manage the disruptions caused by Brexit and Covid-19 are more likely to have high survivability rates and vice versa.

**Table 12:** Correlation Analysis 2

		How has your organisation managed the disruption due to Brexit and Covid-19 pandemic?	Organisation Survivability
How has your organisation managed the disruption due to Brexit and Covid-19 pandemic?	Pearson Correlation	1	
	Sig. (2-tailed)		
	N	14	
Organisation Survivability	Pearson Correlation	.573^*^	1
	Sig. (2-tailed)	.032	
	N	14	14

*Source* Own

*Correlation is significant at the 0.05 level (2-tailed)

#### Regression analysis

Regression analysis enables us to predict one variable based on information about other variables (Wagner III 2009)**.** Two hypotheses are tested using the regression method of inferential statistics.

#### Hypothesis 1

##### H0

The ability of an organisation to manage the disruption caused by Brexit and the Covid-19 pandemic does not have any significant impact on the survivability of the organisation.

##### H1

The ability of an organisation to manage the disruption caused by Brexit and the Covid-19 pandemic has a significant impact on the organisation's survivability.

A linear regression study was performed to see if the ability of firms to manage the disruption induced by Brexit and the Covid-19 outbreak may predict their long-term viability. The hypothesis tests if an organisation's management of the disruptions caused by the Brexit and Covid-19 pandemic (OM) significantly impacts organisational survivability (OS). The dependent variable OS was regressed on predicting variable OM to test hypothesis H_1._ The result shows that OS significantly predicted OM, F (1, 13) = 5.877551, *p* < 0.05, which indicates that OM can play a significant role in shaping OS (b = 0.573, *p* < 0.05). In addition, the R^2^ = 0.329 depicts that the model explains 32.9% of the variance in OS. Table 13 below shows a summary of the findings.

**Table 13:** Summary of Result from Hypothesis 1 testing

Hypothesis	Regression Weights	Beta Coefficient	R^2^	F	*p*-value	Hypotheses Supported
H_1_	OM → OS	0.573	.329	5.877551	0.032	Yes

Source: Own

The result shows that the hypothesis that an organisation's ability to manage the disruption caused by Brexit and Covid-19 pandemic effectively has a significant impact on the survivability of the organisation is supported and the null hypotheses rejected.

#### Hypothesis 2

##### H0

Government's understanding of the problems posed by Brexit and Covid-19 does not have any significant effect on how these events impact organisations.

##### H1

Government’s understanding of the problems posed by Brexit and Covid-19 has an effect on how these events impact organisations.

The study also regressed the outcome variable impact of Brexit and Covid-19 pandemic on an organisation by an independent variable government’s understanding of the problems posed by Brexit and Covid-19. This is an important measure as it helps in understanding whether the government's knowledge of the risks or consequences associated with Brexit and the economic and health implications of the Covid-19 pandemic has any significant impact on organisations. Perhaps, government's appreciation of the potential problems these events may pose could drive the provision of palliatives, bailouts and develop policies that can mitigate the adverse impacts on organisations.

To see if the government's awareness of the difficulties posed by Brexit and Covid-19 (GU) can anticipate the impact it will have on organisations, and a linear regression analysis was done (IO). The dependent variable IO was regressed on predicting variable GU to test hypothesis H_1._ The result shows that GU significantly predicted IO, F(1, 13) = 21.165, *p* < 0.01, which indicates that GU can play a significant role in shaping IO (b = − 0.667, *p* < 0.01). In addition, the R^2^ = 0.638 depicts that the model explains 63.8% of the variance in IO. Table 14 below shows a summary of the findings.

**Table 14:** Summary of Hypothesis 2 results testing

Hypothesis	Regression Weights	Beta Coefficient	R^2^	F	*p*-value	Hypotheses Supported
H_1_	GU → IO	− 0.667	0.638	21.165	0. .001	Yes

*Source* Own

The null hypothesis is rejected, indicating that the hypothesis that the government's awareness of the difficulties posed by Brexit and Covid-19 influences how these high-risk events impact organisations is validated. This implies that to reduce the negative impact of Brexit and Covid-19 on organisations, the government must fully understand the unprecedented economic, social, political, health, and diplomatic challenges associated with both Brexit and Covid-19. Furthermore, the government must develop policies and long-term strategic agendas that will minimise risks and maximise the opportunities to ensure the growth and resilience of organisations operating in the ro-ro freight industry. The following section present further discussion of the analysis.

## Discussion

Data analysis carried out in preceding sections used descriptive and inferential analysis to answer the research question on the issues associated with RoRo ferry industry-level indicators due to Brexit and Covid 19 between the UK, Ireland, and continental Europe. Christodoulou and Kappelin (2020) illustrate in Fig. 2.5 on RoRo shipping competitiveness to be influenced by various factors such as cost, reliability, regularity, transit time and speed, and efficient port operations. However, De Langen et al. (2013) argue that if these level indicators are affected, it will decrease organisation competitiveness. The description of question 3 in Fig. 5.2 of data analysis shows that most organisations were severely and some moderately impacted by Brexit and Covid 19 pandemic. Questions 5 and 6 further determine areas of impact. The researcher developed themes from the respondent responses. Across the themes, the factors for competitiveness indicated by Christodoulou and Kappelin (2020) have been adversely impacted. Nevertheless, by understanding the relationship between variables, Table 5.8 shows that companies who can effectively manage the disruptions caused to these level indicators caused by Brexit and Covid 19 are more likely to have high survivability rate, resulting in the possibility of maintaining an effective RoRo shipping competitiveness. In addition, the collaboration between actors in the RoRo freight movement and government understanding of the impacts of Brexit and Covid 19 is paramount to continuous freight movement, which is tested and summarised in Table 5.10.

## Conclusion

This research focuses on the industry-level analysis of the RoRo ferry market and maritime links between the UK and the EU following Brexit and the Covid-19 pandemic. RoRo ferry service is largely used in the UK, ROI, and the European continent. RoRo firms use maritime links between these nations to operate on the same routes across the countries. Following the impact of Brexit and the Covid 19 pandemic, there have been restrictions and new regulations by the governments which disrupted the supply chain of freight movement. Mixed-method research was used in this research, with qualitative and quantitative gathering and analysis of data. Statistical analysis was used and hypothesis tested. The mixed-method allowed us to research the ferry RoRo market, maritime links, and its survivability amidst the Brexit and Covid crisis between the UK and short sea shipping connections with Ireland and continental Europe. The result shows that the ability of an organisation to manage these disruptions significantly impacts the survivability of the organisation. It also shows that government understanding of Brexit and Covid 19 influences the impact on an organisation. In conclusion, despite Brexit and Covid, the impact on organisations is of moderate effect. However, due to uncertainties, continuous strategies should be put in place with an effective flow of information for continuous freight transport.

This research suggests further research work on a comparative analysis between the direct Roll-on /Roll-off (RoRo) and Lift-on/Lift-off (LoLo) services to the continent. Likewise, due to limited research in the field, continuous study of the RoRo freight market is suggested for future work.

The use of statical analysis allowed the relationship between variables. However, the study was limited due to the small sample size. Most inferential statistical analyses require a large sample size for an accurate representation of variables. On the other hand, correlation and regression are employed in analysis to understand the relationship between the dependent and independent variables. In a future study, the sample group and expected response rate should be considered.

## Data Availability

Available on request.

## Abbreviations

BBC: British broadcasting corporation
BPA: British ports association
Brexit: British exit
Covid: Coronavirus
DfT: Department for transport
ESRI: The Economic and social research institute
EU: Europe
HMRC: Her majesty's revenue and customs
IMDO: Irish maritime development office
ISL: Institute of shipping economics and logistics
NSM: North Sea–Mediterranean corridor
RHA: Road haulage association
ROI: Republic of Ireland
Ro-Ro: Roll on-Roll off
SCM: Supply chain management
SPSS: Statistical package for the social sciences
SSS: Short sea shipping
TENT-T: Trans-European transport network
UK: United Kingdom
